# Potential global geographical distribution of *Lolium temulentum* L. under climate change

**DOI:** 10.3389/fpls.2022.1024635

**Published:** 2022-11-10

**Authors:** Ming Yang, Haoxiang Zhao, Xiaoqing Xian, Hui Liu, Jianyu Li, Li Chen, Wanxue Liu

**Affiliations:** ^1^ School of Life Sciences, Hebei University, Baoding, China; ^2^ State Key Laboratory for Biology of Plant Diseases and Insect Pests, Institute of Plant Protection, Chinese Academy of Agricultural Science, Beijing, China; ^3^ The National Agro-Tech Extension and Service Center, Beijing, China; ^4^ Institute of Plant Protection, Fujian Academy of Agriculture Sciences, Fuzhou, China

**Keywords:** MaxEnt, potential geographical distribution, *Lolium temulentum*, invasive alien plant, climate change

## Abstract

Invasive alien plants posed a significant threat to natural ecosystems, biodiversity, agricultural production, as well as human and livestock health. *Lolium temulentum*, an annual invasive alien weed with fibrous roots, can reduce wheat production and cause economic losses. Moreover, the consumption of grains or cereal products mixed with darnel can cause dizziness, vomiting, and even death. Therefore, darnel is regarded as one of ″the worst weeds around the world″. In the present study, we predicted the potential global geographical distribution of *L. temulentum* using an optimal MaxEnt model, based on occurrence records and related environmental variables. The mean AUC, TSS, and KAPPA were 0.95, 0.778, and 0.75, indicating the MaxEnt model accuracy was excellent. The significant environmental variables, including the mean temperature of coldest quarter (bio 11), precipitation of coldest quarter (bio 19), temperature annual range (bio 7), and annual precipitation (bio 12), produced a great impact on the potential global geographical distribution of *L. temulentum*. Under the current climate, *L. temulentum* was primarily distributed in south-eastern Asia, Europe, and south-eastern North America. The widest total suitable habitat was distributed in Asia, covering nearly 796 × 10^4^ km^2^. By the 2050s, the potential geographical distribution of *L. temulentum* was expected to decrease in the Northern Hemisphere, and shrink gradually in southern America, Africa, and Oceania. Moreover, the distribution center of *L. temulentum* was expected to shift from Asia to Europe. Based on these predictions, changes in the suitable habitats for *L. temulentum* between Europe and Asia warrant close attention to prevent further spread.

## Introduction

Biological invasions, including those by invasive alien plants (IAPs), animals, and microorganisms, have become an increasingly severe issue worldwide, and these phenomena are affected by climate warming (Pimentel et al., 2001; Cornelissen et al., 2019). Furthermore, human activities, including logging, mining, damming, and others, also have a great impact on the distribution of IAPs (Seabloom et al., 2003; MacDougall and Turkington, 2005; Didham et al., 2007). IAPs pose a threat to not only biodiversity and natural ecosystems but also human and livestock health (Vilà et al., 2011). Climate warming and human activities can accelerate population growth and promote the reproduction of IAPs, aggravating their impact on the ecological environment, agricultural economy, and food security (Keller and Shea, 2021). Reportedly, IAPs were responsible for estimated annual economic losses of US$27.9, US$1.4, US$2.4, US$1.5, US$37.8, and US$17 billion in the crop sector in the United States, United Kingdom, Australia, South Africa, India, and Brazil, respectively (Pimentel et al., 2001). Poaceous plants are regarded as important IAPs and have been listed as quarantine plants in many countries. For instance, according to ″The U. S. Regulated Plant Pest List″, 40 Poaceous IAPs have been regarded as quarantine targets. Additionally, 41 Poaceous IAPs were listed in the ″Catalogue of Quarantine Pests for Import Plants to The People’s Republic of China″. *Lolium temulentum* is one of the critical Poaceous IAPs. At present, *L. temulentum* has been included in the entry quarantine list of China, Latvijas-Republika, and parts of the United States, including Florida, Alabama, Arkansas, Mississippi, Georgia, Louisiana, Oklahoma, South Carolina, Tennessee, and Texas.

*Lolium temulentum*, or darnel, is an invasive annual weed worldwide, regarded as one of ″the worst weeds worldwide″ and a competitive C3 grass weed that typically grows with wheat, sunflowers, and other winter crops under temperate climates (Lush, 1976; Holm et al., 1977; Angiras and Modgal, 1981; Sarno et al., 1986; Helm et al., 1987). The germination and growth of *L. temulentum* required low temperature and high soil moisture conditions (Steiner and Ruckenbauer, 1995). *Lolium temulentum* was also generally regarded as a competitive IAP. For instance, *L. temulentum* bears a fibrous root system that enables better nutrient absorption than other cereals (Angiras and Modgal, 1981). Consequently, darnel seeds often intermix with wheat during harvest, and the mixed seeds are difficult to separate from cereal grains (Cousland, 2015). Therefore, darnel can easily spread throughout the world *via* global wheat trade. *Lolium temulentum* is native to the Mediterranean and southwestern Asia and is widely distributed in Europe, southern Africa, eastern Asia, and North America (Terreix, 1968; Holm et al., 1977). In early 1942, *L. temulentum* seeds were reported to exert toxicity in humans and other non-human animals when consumed in conjunction with wheat in North America (Cousland, 2015). In Asia and Africa, grain stocks with plentiful darnel were reported to cause dizziness, vomiting, coma, and even death in humans and livestock (Rizk and Hussiney, 1991; Musselman, 2000). In addition, field experiments in North America demonstrated that high Persian darnel (*Lolium persicum*) densities reduced spring wheat and sunflower yield by up to 83% and 57%, respectively, causing severe agricultural economic losses (Holman et al., 2004). In addition, *L. temulentum* can serve as a host to the fungus, crop pathogens, and parasitic nematodes, such as *Puccinia striiformis* (Zhukova and Kupriyanova, 1981; Ibrahim et al., 1988). Previous studies on *L. temulentum* were focused on plant biology, such as molecular genetics, phylogeny, and plant pathology (Thomas et al., 2011). However, the global distribution pattern of *L. temulentum* remains unknown. Therefore, predicting the potential geographical distribution of *L. temulentum* is fundamental for its monitoring and control worldwide, and can build a foundation for the management of other critical IAPs.

Species distribution models (SDMs), including maximum entropy (MaxEnt), CLIMEX, genetic algorithm for rule-set prediction, and Biomod2, have been widely used to predict the potential geographical distribution of species under climate change (Phillips et al., 2004; Willis and Bhagwat, 2009; Thuiller et al., 2014). Based on the maximum entropy theory, MaxEnt model makes predictions with presence data *via* the machine learning response (Phillips et al., 2006; Favretti, 2018). MaxEnt model offers certain advantages over other SDMs, such as support for small sample sizes, high complexity, and better model performance (Xiong et al., 2004). More importantly, the MaxEnt model is more robust for spatial errors to predict species distribution with occurrence data and presence-only datasets (Graham et al., 2008; Convertino et al., 2014). Therefore, this model has been widely applied to predict the potential geographical distribution of IAPs under the current climate and future climate change, such as *Paspalum distichum* in the Korean Peninsula and *Sorghum almum* worldwide (Phillips et al., 2004; Lee et al., 2016; Wang and Wan, 2020). However, there are also some weaknesses in the MaxEnt model that requires further study, including the regularization of avoiding overfitting and fewer methods in estimating prediction of the amount of error (Phillips et al., 2004; Phillips et al., 2006). In previous studies, ENMevals has been commonly used to calibrate the MaxEnt model, which can be ″tuned″ to generate optimal model complexity and avoid model overfitting (Muscarella et al., 2014a; Radosavljevic and Anderson, 2014; Roberts et al., 2017; Hallgren et al., 2019). Based on the running environment of R package, ENMevals across k-fold cross-validation can get partitioning data that can be used to select the optimal MaxEnt model settings for users’ different demands (Muscarella et al., 2014). Therefore, in the present study, we used an optimal MaxEnt model to predict the potential global geographical distribution of *L. temulentum.*

Owing to the biological characteristics of high adaptability, potential reproduction, and toxicity, *L. temulentum* could establish population quickly in new habitat mixed with wheat, leading to cereal loss and food poisoning for human and livestock. Therefore, predicting the potential global geographical distribution of *L. temulentum* is significant for, agricultural production and human health. In the present study, we aimed to predict the potential global geographical distribution of *L. temulentum* under the current and future climates using the optimal MaxEnt model. First, significant environmental variables affecting *L. temulentum* distribution were analyzed. Next, the potential geographical distribution of *L. temulentum* was predicted using the optimal MaxEnt model under the current climate and future climate scenarios in the 2050s. Finally, changes in the potential geographical distribution of *L. temulentum* and distribution center shifts between the current climate and future climate change were analyzed. To the best of our knowledge, the present study is the first to predict the potential geographical distribution of *L. temulentum*, which will facilitate the surveillance and control of IAP in the future.

## Material and methods

### Species occurrence records

**Figure 1:**
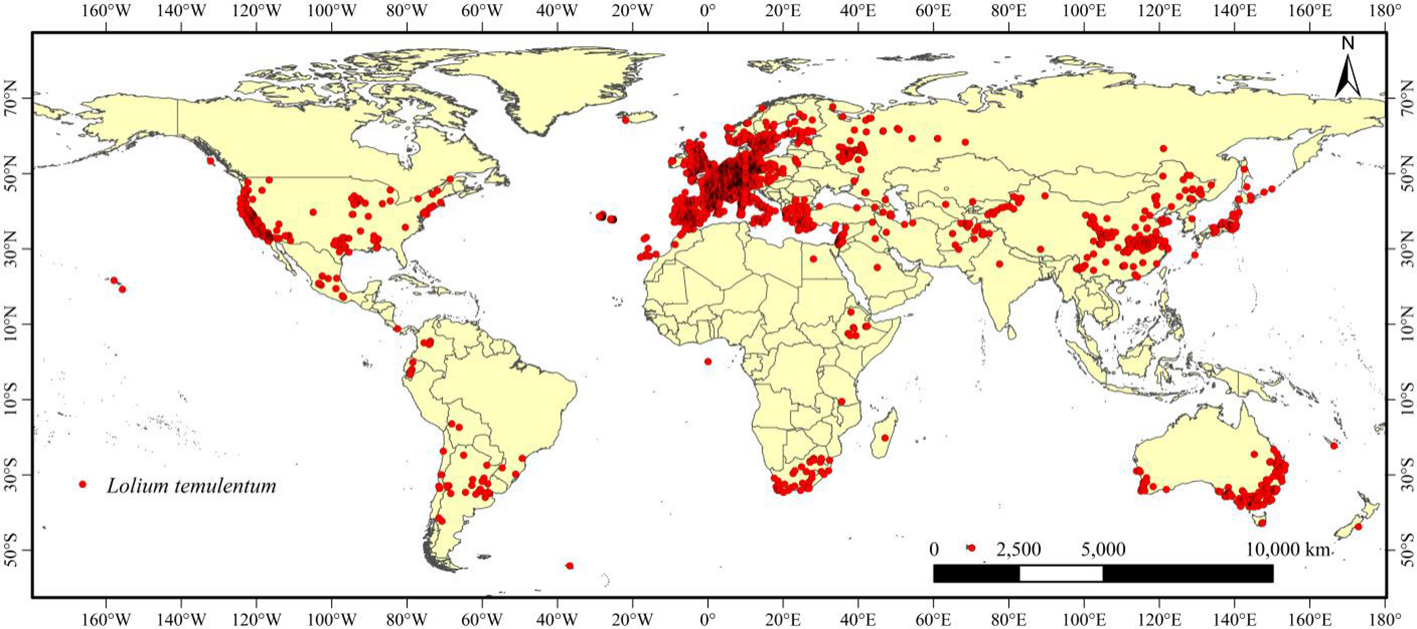
Global distribution points for *Lolium temulentum*.

We obtained occurrence records of *L. temulentum* from online and literature databases, including 5,059 records from the Global Biodiversity Information Facility (GBIF: http://www.gbif.org), 117 records from published articles in the China National Knowledge Infrastructure (CNKI: https://www.cnki.net ), 20 records from the Chinese Virtual Herbarium (CVH: https://www.cvh.ac.cn ), and 80 records from the Ministry of Agriculture and Rural Affairs of the People’s Republic of China (http://www.moa.gov.cn/ ). A total of 5,276 occurrence records of *L. temulentum* collected from across the world were obtained, longitude and latitude of occurrence records in each continent were shown in supplementary excel. ArcGIS was used to map the global occurrences of *L. temulentum*. We used ENMTools to screen the occurrence records and ensure that only a single occurrence record was retained within each 10 × 10 km^2^ raster (Warren et al., 2010). Finally, 3,899 occurrence records were obtained worldwide (**Figure 1**).

### Environmental variables

Nineteen environmental bioclimatic variables during 1979–2013 at 5-arc minute resolution were downloaded from the Paleoclim database (http://www.paleoclim.org// ) (Brown et al., 2018). Future climate and elevation (altitude) data (5-arc minute resolution) were downloaded from the WorldClim database (https://www.worldclim.org/ ). Since the 2050s (2040-2060) is the closest period to the current (1979–2013), we aimed to provide time-sensitive references for the prevention and control of *L. temulentum* worldwide. Therefore, the future climate data selected the four representative concentration pathways (RCP 2.6, RCP 4.5, RCP 6.0, and RCP 8.5) in the 2050s (2040–2060) using the Community Climate System Model (CCSM) 4.0. Slope and Aspect were extracted from a digital elevation model using ArcGIS (Flessner et al., 2021). Soil variables were selected from the Harmonized World Soil Database v1.2 of the United Nations Food and Agriculture Organization (https://www.fao.org/soils-portal/data-hub/soil-maps-and-databases/harmonized-world-soil-database-v12/en/ ) (Chefaoui et al., 2016). The 27 environmental variables were listed in **Table S1**.

Linear correlations among the environmental variables lead to overfitting of the MaxEnt model (Graham, 2003). Therefore, we used EMNTools to eliminate the multivariate collinearity of the 27 environmental variables (Warren et al., 2010) (**Figure S1**). When two environmental variables were strongly correlated (|r|≥0.8), the more meaningful variable was retained. Finally, 13 environmental variables were considered (**Table 1**).

**Table 1:** Percent contribution and permutation importance of environmental variables to the potential geographical distribution of *Lolium temulentum*.

Variable	Description	Unit	Percent contribution	Permutation importance
bio2	Mean diurnal temperature range	°C	44.3	71.2
bio7	Temperature annual range	°C	26.1	4.3
bio11	Mean temperature of coldest quarter	°C	16.7	7.6
bio12	Annual precipitation	mm	8.2	10.1
bio15	Precipitation seasonality (coefficient of variation × 1)	–	1	2.1
bio17	Precipitation of driest quarter	mm	0.8	0.5
bio19	Precipitation of coldest quarter	mm	0.8	1.6
Altitude	Altitude	m	0.8	0.5
Slope	Slope		0.6	0.7
ADD_PROP	Other properties (gelic, vertic, petric)		0.5	0.4
T_OC	Topsoil Organic Carbon	% wt	0.3	0.9
T_PH_H_2_O	Topsoil pH (H_2_O)	-log(H^+^)	0.1	0.1
T_SAND	Topsoil Sand Fraction	% wt	0.1	0.1

### Model calibration, settings, and evaluation

Regularization multiplier (RM) and feature combinations (FCs) were the key parameters of the MaxEnt model, which were calibrated to increase prediction accuracy (Muscarella et al., 2014). Based on the Pearson correlation coefficient, the ENMeval package in R version 4.1.3 was used to calibrate RM and FCs, and the output ‘excel’ files were used to select different parameter settings (RM and FCs) for the optimal MaxEnt. RM was set from 0.5 to 4 with an interval of 0.5, based on the occurrence records of *L. temulentum* and environmental variables. MaxEnt model included five FCs (i.e., linear-L, quadratic-Q, hinge-H, product-P, and threshold-T) and their six combinations (i.e., H, L, LQ, LQH, LQHP, and LQHPT) (Phillips and Dudík, 2008). For the 48 combinations, we selected the minimum values of delta Akaike information criterion correction (ΔAICc) using the ENMeval package in RStudio (Akaike, 1992). Based on the lambdas file, when ΔAICc was 0, RM was set to 0.5, and FC was set as LQHPT, which was the optimal combination for the MaxEnt model in the present study (Warren and Seifert, 2011) (**Figure S2**).

In the present study, 3,899 occurrence records and 13 bioclimatic variables were imported into MaxEnt 3.4.4. using the ″dismo″ package in R version 4.1.3. Of all the occurrence records, 75% were selected as the training data and 25% as the testing data. The FC was set as LQHPT, with 0.5 RM and 10 replicates. The maximum number of iterations was set to 500, with a maximum of 10000 background points. Random seeds were assessed to increase model randomness. The replicated run type was set as ″Bootstrap″ and the other parameters were set as default. The receiver operating characteristic (ROC) curve and area under the ROC curve (AUC) were used to evaluate model accuracy (Roca et al., 2004). Besides, true skill statistics (TSS) and KAPPA statistics values were considered as evaluated criteria. AUC was a metric based on the rates of both true positive and true negative. The AUC value ranged from 0 to 1, divided into four grades based on the evaluation criterion of accuracy: poor (0.5<AUC<0.7), moderate (0.7<AUC<0.9), and excellent (AUC>0.9) (Franklin and Miller, 2010). TSS was a dependent threshold depending on sensitivity and specificity (Allouche et al., 2006). KAPPA evaluated the match of model predictions and the truth, controlling the random accuracy *via* the expected accuracy (Cohen, 1960). The higher the TSS and KAPPA values, the higher the accuracy of the MaxEnt model outputs

The modeling results generated distribution predictions in a grid raster format with indices ranging from 0 to 1 (Ceylan and Gül, 2022). The potential geographical distribution of *L. temulentum* was classified into four categories under the current climate and future climate change scenarios in the 2050s: unsuitable habitat (P<0.34), poorly suitable habitat (0.34≤P<0.5), moderately suitable habitat (0.5≤P<0.7), and highly suitable habitat (0.7≤P<1) based on the Maximum training sensitivity plus specificity cloglog threshold of the MaxEnt model (Cantor et al., 1999; Manel et al., 2001).

### Suitable habitat index classification and distribution center shift

ArcGIS was used to analyze the distribution center shifts of *L. temulentum* during the current climate and future climate change scenarios in the 2050s. The initial potential geographical distribution of *L. temulentum* was reclassified into two categories: unsuitable (P<0.34) and suitable (0.34≤P<1) habitat. Floating point data of the potential geographical distribution were regarded as the weight to combine the spatial location of the floating point for calculating the centroid. The centroid trends were used to analyze the changes in the potential geographical distribution of *L. temulentum* between the current and future climates.

### Multivariate environmental similarity surface

Environment has been verified as the critical variable for structuring species distributions. However, spatial autocorrelation should be considered a major aspect of the interplay in both environmental variables and geographic space (Elith and Leathwick, 2009). Multivariate environmental similarity surface (MESS) was used to analyze the similarity (S) between current and future climates. If S < 0, which is regarded as climate anomaly, and S = 100, which indicates that the future climate is identical to the current. The increasingly negative values mean greater dissimilarity so that reduced model transferability (Ranjitkar et al., 2016). It is likely to lead to geographic species clumping because of the impact of the environmental layer or geographic space. In the present study, we calculated the similarity both the current and future layers including RCP 2.6, RCP 4.5, RCP 6.0, and RCP 8.5 through density. tool. novel in the MaxEnt model for predicting the potential global geographical distribution of *L. temulentum*.

## Results

### Model evaluation and significant environmental variables

The mean AUC, TSS, and Kappa values of the optimal MaxEnt model were 0.95 (± 0.001), 0.78 (± 0.01), and 0.75 (± 0.01), indicating the model accuracy was reliable (**Figure S3**). The importance of the environmental variables were evaluated according to their percent contribution rates and the ″Jackknife method″ (**Figure S4**, **Table 1**). Specifically, mean temperature of coldest quarter (bio 11), precipitation of coldest quarter (bio 19), temperature annual range (bio 7), and annual precipitation (bio 12) were significant environmental variables affecting the potential geographical distribution of *L. temulentum*.

We selected a suitable range of environmental variables for *L. temulentum* distribution using the threshold for highly suitable habitats (>0.5). The suitable ranges of mean temperature of coldest quarter (bio 11), temperature annual range (bio 7), precipitation of coldest quarter (bio 19), and annual precipitation (bio 12), were -8.5 to 13.8°C, -2.6 to 34, and 44 to 52.8°C, 60 to 888.3 mm, and 292 to 1,560 mm, respectively (**Figure S5**). The highest values of bio11, bio7, bio19, and bio12 were 3°C, 52.8°C, 778 mm and 578 mm, respectively.

### Potential geographical distribution under the current climate

**Figure 2:**
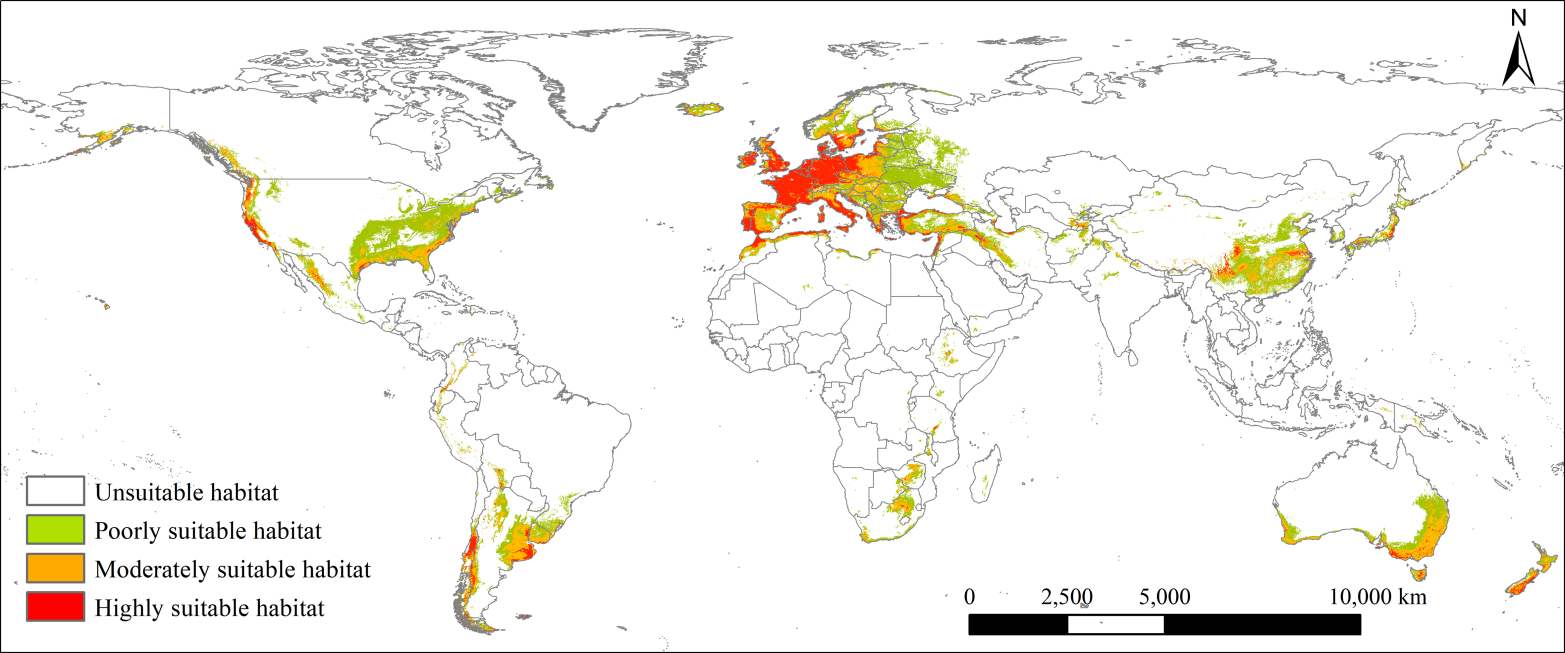
Current potential global geographical distribution of *Lolium temulentum*.

**Figure 3:**
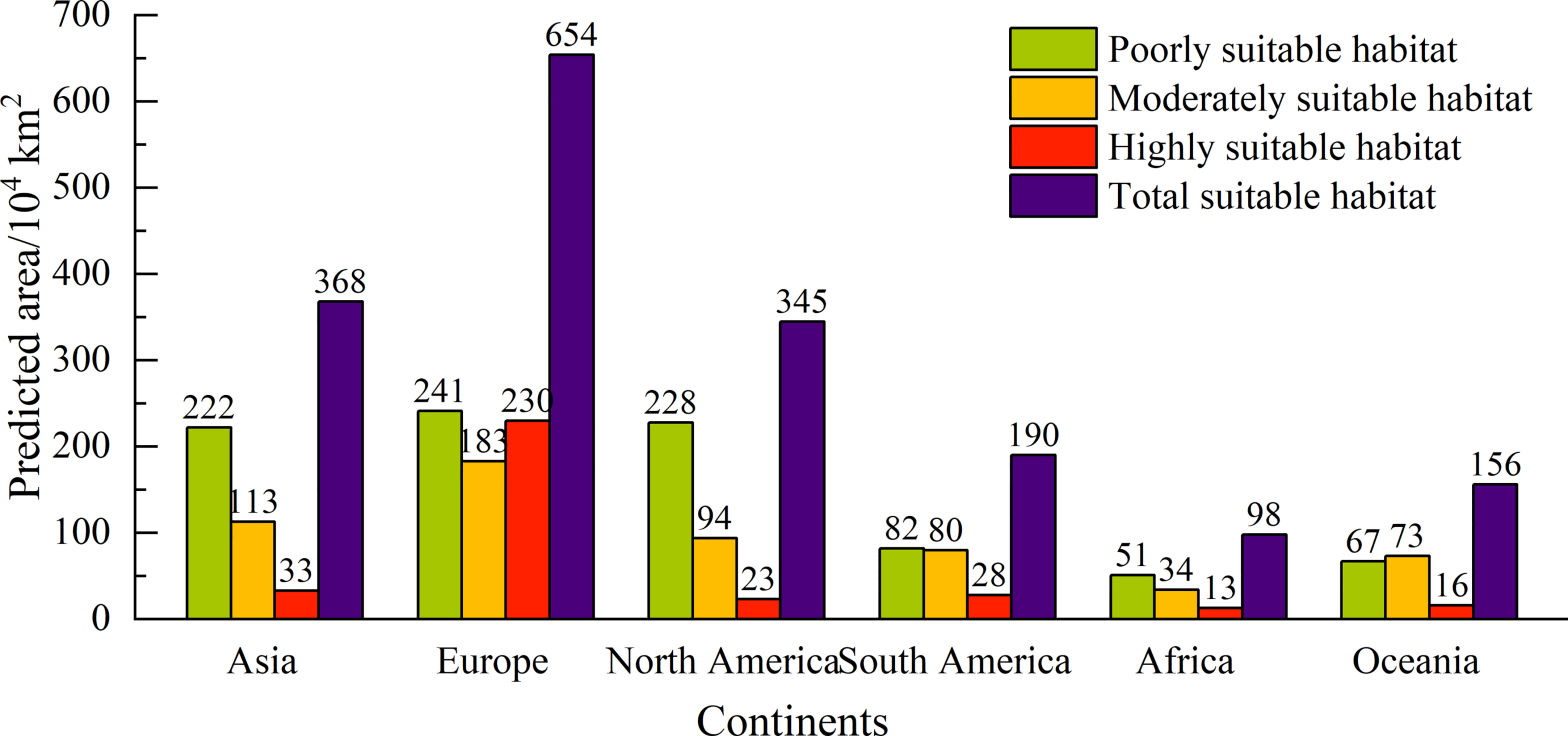
Different types of suitable areas for *Lolium temulentum* across six continents under the current climate.

Under the current climate, the potential global geographical distribution of *L. temulentum* was mainly concentrated in Asia, Europe, North America, and South America (**Figure 2**). The global total, highly, moderately, and poorly suitable habitat areas of *L. temulentum* measured 1811 × 10^4^, 343 × 10^4^, 577 × 10^4^, and 891 × 10^4^ km^2^ respectively (**Figure 3**). The largest total, highly, moderately, and poorly suitable habitat area was located in Europe (654 × 10^4^, 230 × 10^4^, 183 × 10^4^, and 241 × 10^4^ km^2^), including the United Kingdom, Ireland, Jersey, France, Dermark, Germany, Italy, Greece, Portugal, Spain, Andorra, and western Poland; followed by in Asia (368 × 10^4^, 33 × 10^4^,113 × 10^4^, and 222 × 10^4^ km^2^), including southwestern and southeastern China, southeastern Japan, northern Iraq, and Turkey; in North America (345 × 10^4^, 23 × 10^4^, 94 × 10^4^, and 228 × 10^4^ km^2^), including western and southeastern the United States, in South America (190 × 10^4^, 28 × 10^4^, 80 × 10^4^, and 82 × 10^4^ km^2^), including Chile, Uruguay, and southern Argentina; in Africa (98 × 10^4^, 13 × 10^4^, 34 × 10^4^, and 51 × 10^4^ km^2^), including northern Morocco, northern Algeria, Mozambique, and Swaziland; in Oceania (156 × 10^4^, 16 × 10^4^, 73 × 10^4^, and 67 × 10^4^ km^2^), including New Zealand, southwestern and southeastern Australia.

### Future potential global geographical distribution

**Figure 4:**
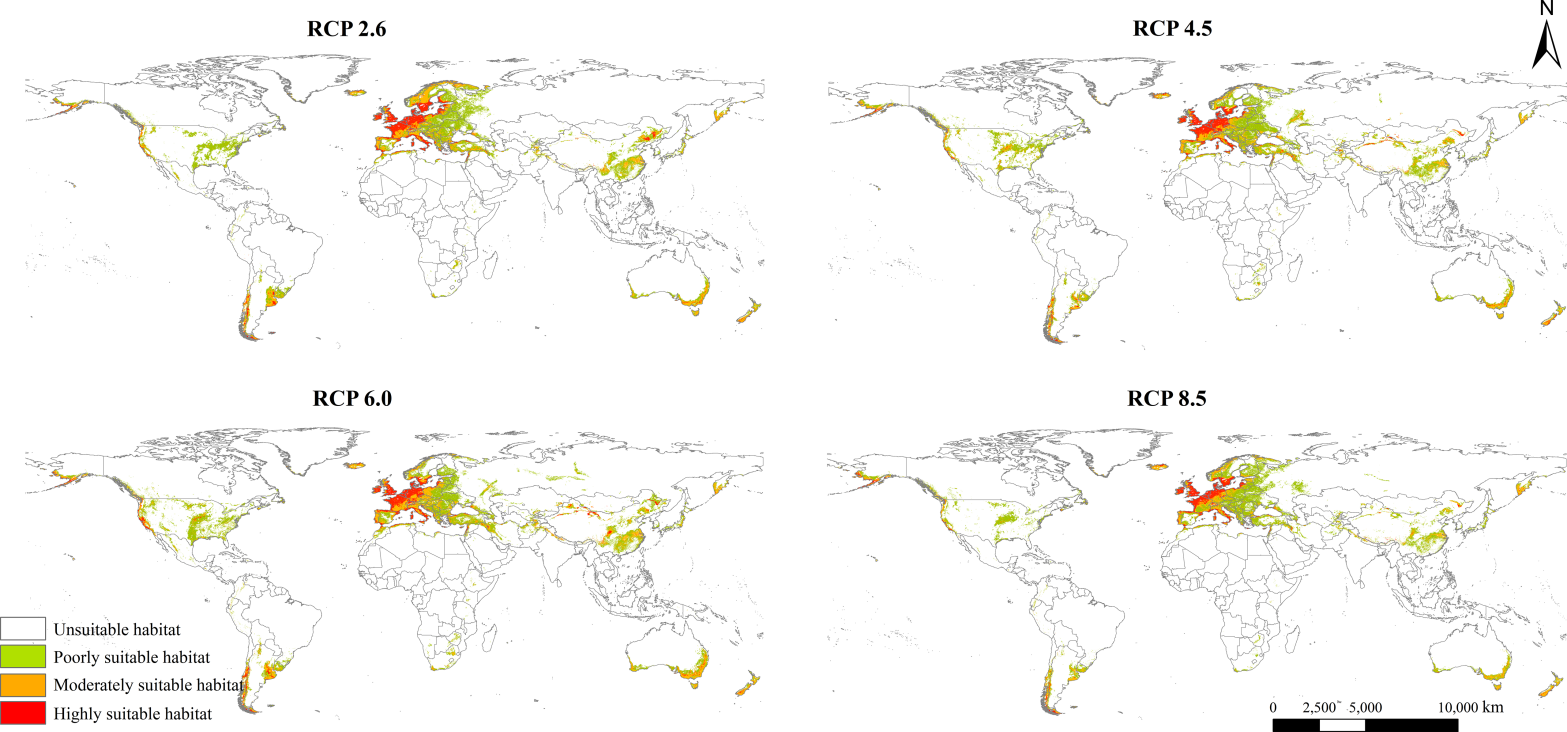
Potential global geographical distribution of *Lolium temulentum* under four climate scenarios in the 2050s, namely RCP 2.6, RCP 4.5, RCP 6.0, and RCP 8.5.

The potential geographical distribution patterns of *L. temulentum* in the 2050s under four different scenarios, namely RCP 2.6, RCP 4.5, RCP 6.0, and RCP 8.5, were presented in **Figure 4**. Compared with those under the current climate, the total suitable areas of *L. temulentum* in each continent shrank except in Europe. Moreover, the highly, moderately, and poorly suitable habitat areas of *L. temulentum* reduced to different extents (**Figure S6**; **Table S2**). the potential geographical distribution of *L. temulentum* was primarily concentrated in almost all of Europe, southeastern Asia, and southeastern North America.

The total, moderately, and poorly suitable areas of *L. temulentum* in Asia increased under RCP 6.0 in the 2050s, and other scenarios decreased. Under RCP 6.0, the total suitable habitat area of *L. temulentum* would increase by 448 × 10^4^ km^2^, reaching the maximum, and this area would be the minimum under RCP 8.5. The total, moderately, and poorly suitable areas of *L. temulentum* in Europe increased under RCP 6.0 in the 2050s. Under RCP 2.6, the total suitable habitat area of *L. temulentum* would increase by 796 × 10^4^ km^2^, reaching the maximum, and this area would be the minimum under RCP 6.0. The highly suitable areas of *L. temulentum* in North America increased under RCP 6.0 in the 2050s, and other scenarios decreased. Under RCP 6.0, the total suitable habitat area of *L. temulentum* achieved the minimum under RCP 8.5. In South America, under RCP 8.5, the total suitable habitat areas of *L. temulentum* would decrease by 94 × 10^4^ km^2^, dropping to the minimum, and this area would be the maximum under RCP 6.0. In Africa, under RCP 8.5, the total suitable habitat area of *L. temulentum* would decrease by 26 × 10^4^ km^2^, dropping to the minimum, and this area would be the maximum under RCP 6.0. In Oceania, under RCP 8.5, the suitable habitat area of *L. temulentum* would decrease by 85 × 10^4^ km^2^, dropping to the minimum, and this area would be the maximum under RCP 6.0.

### Changes in potential global geographical distribution and distribution center shifts

**Figure 5:**
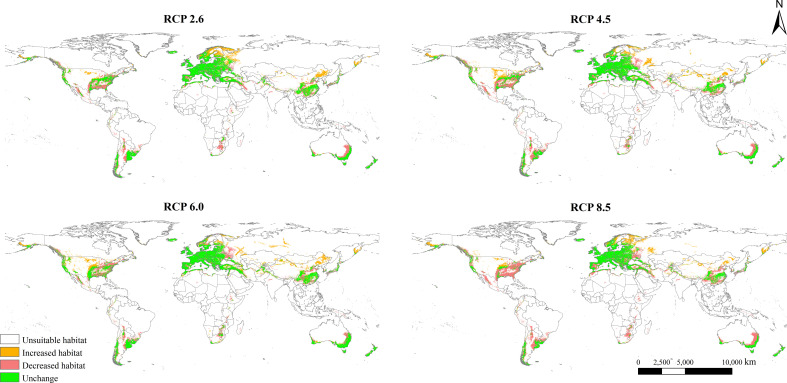
Spatial changes in *Lolium temulentum* distribution under future climate scenarios, including RCP 2.6, RCP 4.5, RCP 6.0, and RCP 8.5.

Changes in the potential geographical distribution of *L. temulentum* under the four scenarios in the 2050s were shown in **Figure 5**. The global total suitable area of *L. temulentum* would increase by 396 × 10^4^, 410 × 10^4^, 449 × 10^4^, and 370 × 10^4^ km^2^ under RCP 2.6, RCP 4.5, RCP 6.0, and RCP 8.5, respectively, being concentrated in northeastern and northwestern China, southeastern Russia, and Turkey in Asia, Sweden, Finland in Europe, southeastern the United States in North America. Meanwhile, the global total suitable area of *L. temulentum* would decrease by 549 × 10^4^, 622 × 10^4^, 526 × 10^4^, and 782 × 10^4^ km^2^ under RCP 2.6, RCP 4.5, RCP 6.0, and RCP 8.5, respectively, being concentrated in southern China, Japan, Iran in Asia, western Russia, Ukraine, Spain in Europe, southeastern the United States and Mexico in North America, Bolivia, Argentina in South America, Morocco and South Africa in Africa, southwestern and southeastern Australia in Oceania.

**Figure 6:**
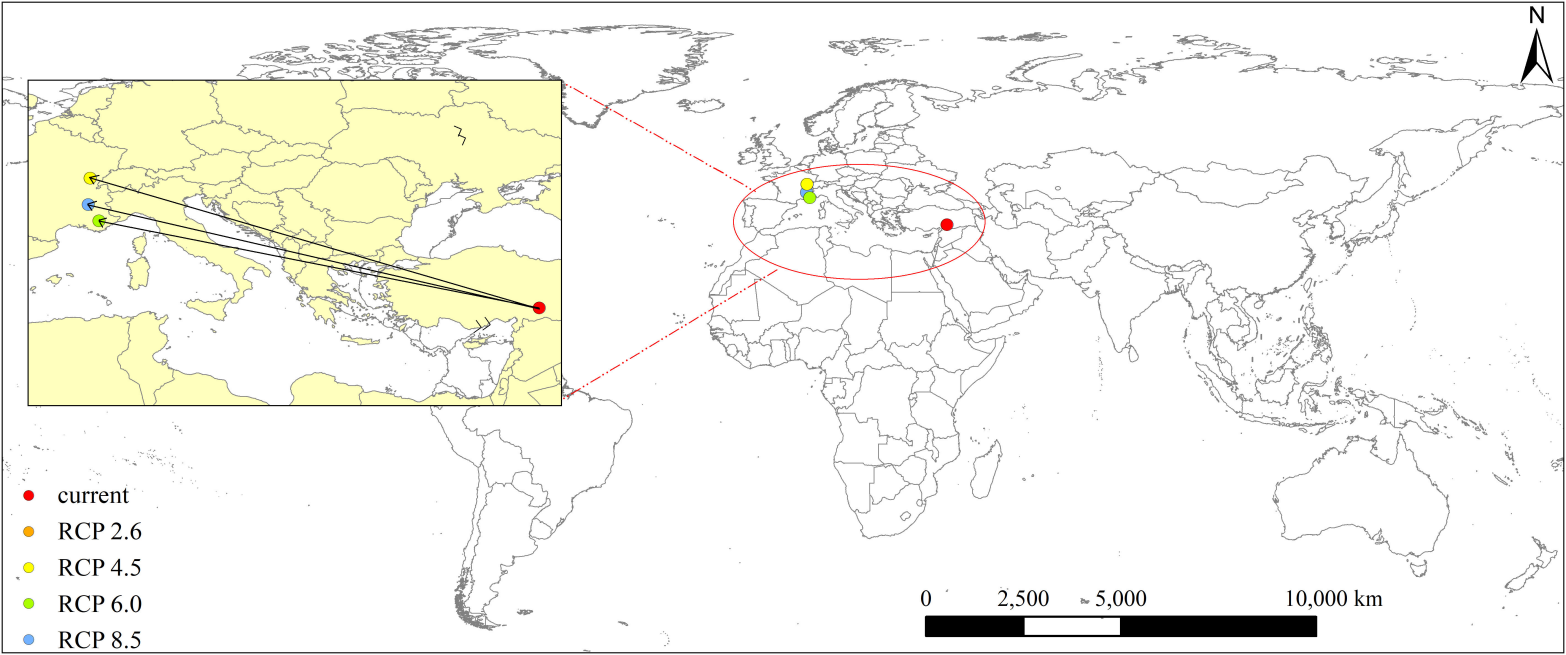
Distribution center shifts of *Lolium temulentum* under the current and future climate scenarios.

Under the current climate, the distribution center of *L. temulentum* was located in Turkey (37° 43′ 04″ E, 37° 58′ 40″ N) (**Figure 6**). In the 2050s, under the RCP 2.6 (RCP 4.5), RCP 6.0, and RCP 8.5, the distribution center of *L. temulentum* would transfer to France (5° 24′ 50″ E, 46° 57′ 00″ N) or (5° 16′ 11″ E, 45° 1′ 33″ N), and Monaco (6° 11′ 34″ E, 43° 53′ 42″ N), respectively. The distribution center approximately overlapped between RCP 2.6 and RCP 4.5. These shifts differed among RCP 2.6, RCP 6.0, RCP 4.5, and RCP 8.5, indicating that different climate conditions affect the potential geographical distribution of *L. temulentum*. The distribution center of *L. temulentum* transferred 2824.91, 2732.31, and 2838.06 km from the current climate to RCP 2.6 (RCP 4.5), RCP 6.0, and RCP 8.5. Most notably, under the future four scenarios, the spread trends of distribution centers were transferred to the high-latitudes of the world compared to the current climate.

### Analysis of the multivariate environmental similarity surface

**Figure 7:**
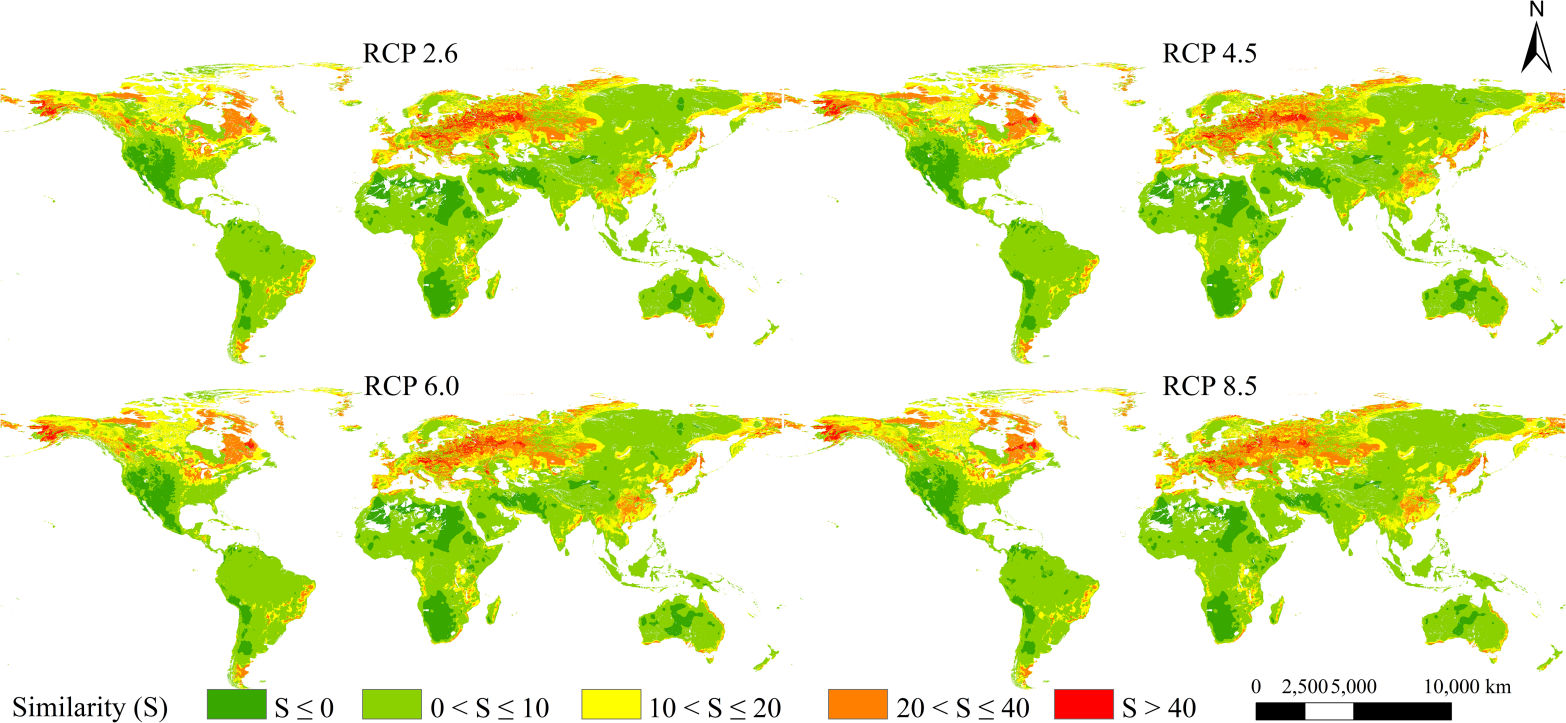
MESS analysis in future four scenarios.

Based on the MESS analysis, the areas of climate anomaly were mainly located in western Asia, western North America, southwestern South America, northern and southern Africa, and southwestern Oceania (**Figure 7**). Under the future scenarios in the 2050s, the mean S values of 3,899 occurrence records of *L. temulentum* worldwide were 15.00 under RCP 2.6, 13.98 under RCP 4.5, 14.48 under RCP 6.0, and 13.80 under RCP 8.5, which indicated that the climate anomaly of RCP 8.5 was greater than other scenarios.

## Discussion

To our best knowledge, the present study is the first to investigate the potential global geographical distribution of *L. temulentum* under the current climate and four distinct future climate scenarios in the 2050s. The default MaxEnt model has the problem of overfitting due to sampling bias. To avoid this issue, we used the ″ENMeval″ package to optimize the MaxEnt model, which was a widely applied approach in previous studies on the potential geographical distribution of IAPs, such as *Panicum maximum*, *Spartina alterniflora*, *Paspalum distichum*, among others (Lee et al., 2016; Liu et al., 2019; Wang and Wan, 2020). *Lolium temulentum*, a significant IAP, with high adaptability and toxicity, can reduce wheat yield and cause food toxicosis (Wilson, 1873). Furthermore, *L. temulentum* has been listed in the quarantine catalog of some countries, because of its adverse effects on the agricultural economy and human health. Therefore, the potential geographical distribution of *L. temulentum* under the current and future climate change conditions must be explored.

*Lolium temulentum* is an annual weed that primarily grows, together with winter crops, and prefers low temperatures (Helm et al., 1987). Our results indicated that mean temperature of coldest quarter (bio11) and precipitation of coldest quarter (bio 19) were the key factors affecting the potential global geographical distribution of *L. temulentum*. Previous studies have shown that *L. temulentum* seeds were dormant at chilling temperatures (2°C) (Ougham, 1987). With rising temperatures, herbicide-mediated *Lolium* spp. control became ineffective, and plant defense mechanisms were promoted when *Lolium* species were growing at 10 to 30 °C (Mucheri et al., 2020). For instance, alkaloids involved in stress resistance were detected in *L. perenne* when temperatures rise above 23°C (Huizing et al., 1991). The above findings all indicated that temperature variables were the most significant variables affecting the potential geographical distribution of *L. temulentum*. Precipitation was found to be another significant environmental variable affecting the growth of *Lolium* species. Previous studies indicated that the suitable range of mean annual rainfall was from 400 to 1200 mm (CABI, 2022). Our findings showed that 578 mm was the optimal value of annual precipitation for the growth of *L. temulentum*. Our findings are consistent with the above findings. Seasonal precipitation increases with climate warming, particularly in winter and summer, in the Southern and Northern Hemispheres, indicating that the wet-season tends to be wetter and dry seasons become slightly drier (Chou et al., 2007). Soil moisture interacted with soil type and depth to affect the development of *L. rigidum* seeds (Narwal et al., 2008). However, this was not a significant variable for *L. temulentum* growth because of its high adaptability.

Furthermore, our results showed that with climate warming, the total suitable areas of Northern Hemisphere were decrease, and those in the Southern Hemisphere gradually shrank. Previous studies on IAPs, such as *Salvinia molesta* and *Eichhornia crassipes*, have shown that the total suitable habitat areas tend to decrease under the RCP 4.5 in the 2050s and 2070s (Kariyawasam et al., 2021). Studies on Poaceous plants have shown that their potential geographical distribution has different trends based on different ecological characteristics. For instance, *Sorghum halepense* is distributed in western Europe, southern North America, and southern South America (Wang and Wan, 2020). Meanwhile, *Spartina alterniflora* prefers areas along the coast of western Europe, southwestern North America, and southwestern Asia (Liu et al., 2019). Furthermore, *Panicum maximum* prefers areas in the Southern Hemisphere, primarily southeastern South America and eastern Africa (Wang and Wan, 2020). The Northern Hemisphere is likely to be warmer than the Southern Hemisphere because of northward cross-equatorial ocean heat transport (Croll, 2012; Kang et al., 2015). The distribution center of *L. temulentum* tended to transfer the high-latitudes in the future scenarios compared with the current climate, mainly from Turkey to France or Monaco. However, a previous study on *Alternanthera philoxeroides* and *Ageratina adenophora* showed that their distribution centers exhibit different trends, presenting shifts to the middle latitudes of the Chinese mainland in the north and west (Tu et al., 2021). Such different expanding tendencies of *L. temulentum* suggest its high adaptability to new environments and transfer to new directions under the four scenarios in the 2050s.

The global dispersal of *L. temulentum* can be attributed to the international wheat trade and human activities. It initially spread to North America and subsequently to Asia. Our results indicated Europe, Asia, and North America as the three major invasion areas under the current and future scenarios in the 2050s. Northeastern and northwestern China, southeastern Russia, and Turkey in Asia, Sweden, Finland in Europe, and southeastern the United States in North America were indicated as new areas of invasion in the 2050s with climate warming. Therefore, we put forward the following prevention and control strategies to prevent the further spread of the IAP, (1) implementation of stringent plant quarantine measures, particularly for imported cereal, flax, and soil associated with the introduced plants; (2) application of agricultural control, such as long rotations, soil disturbance, and high fertilizer applications (Ferrari et al., 1984); and (3) application of chemical control, such as the use of triallate or metoxuron at pre-sowing (Gad and El-Mahde, 1972; Stevens and Meyes, 1976).

## Conclusions

MaxEnt model is widely used to predict the potential geographical distribution with presence data *via* the machine learning response. Compared with other SDMs, MaxEnt model offers certain advantages, including support for small species occurrence, high complexity, better model performance, and more robust for spatial errors to predict species distribution with occurrence data and presence-only datasets. In the present study, we used an optimal MaxEnt model to predict the potential global geographical distribution of *L. temulentum* under the current and four future climate scenarios in the 2050s. Mean temperature of coldest quarter, precipitation of coldest quarter, temperature annual range, and annual precipitation were found to be important environmental variables affecting the further spread of *L. temulentum*. Under the current climate, *L. temulentum* is primarily distributed in southeastern Asia, almost all of Europe, and southeastern and southwestern North America along the coast, although further spread in northwestern Asia, southeastern Europe, and northwestern and southeastern North America were expected in the 2050s. More attention should be paid to changes in the potential geographical distribution of *L. temulentum* to prevent its further spread in Russia, China, Finland, Sweden, and the United States.

## Data availability statement

Publicly available datasets were analyzed in this study. This data can be found here: DOI10.15468/dl.z92v8t.

## Author contributions

MY and HZ and: conception and design of the research. MY: acquisition of data. MY: analysis and interpretation of data. MY and HZ: statistical analysis. HZ: drafting the manuscript. XX, HL, JL, LC and WL: manuscript revision. All authors contributed to the article and approved the submitted version.

## Funding

This work was supported by the National Key R&D Program of China (2021YFC2600400), Technology Innovation Program of Chinese Academy of Agricultural Sciences (grant no. caascx-2017-2022-IAS), and Key R&D Program of Yunnan province (202103AF140007)

## Conflict of interest

The authors declare that the research was conducted in the absence of any commercial or financial relationships that could be construed as a potential conflict of interest.

## Publisher’s note

All claims expressed in this article are solely those of the authors and do not necessarily represent those of their affiliated organizations, or those of the publisher, the editors and the reviewers. Any product that may be evaluated in this article, or claim that may be made by its manufacturer, is not guaranteed or endorsed by the publisher.
