# Supplementary material for: Potential global geographical distribution of Lolium temulentum L. under climate change
Source: Front Plant Sci. 2022 Nov 10;13:1024635. doi: 10.3389/fpls.2022.1024635 (PMC9686299; doi:10.3389/fpls.2022.1024635)
Supplement: Supplementary file 1 [file DataSheet_1.docx]

Supplementary Material

# Supplementary Figures and Tables

## Supplementary Figures


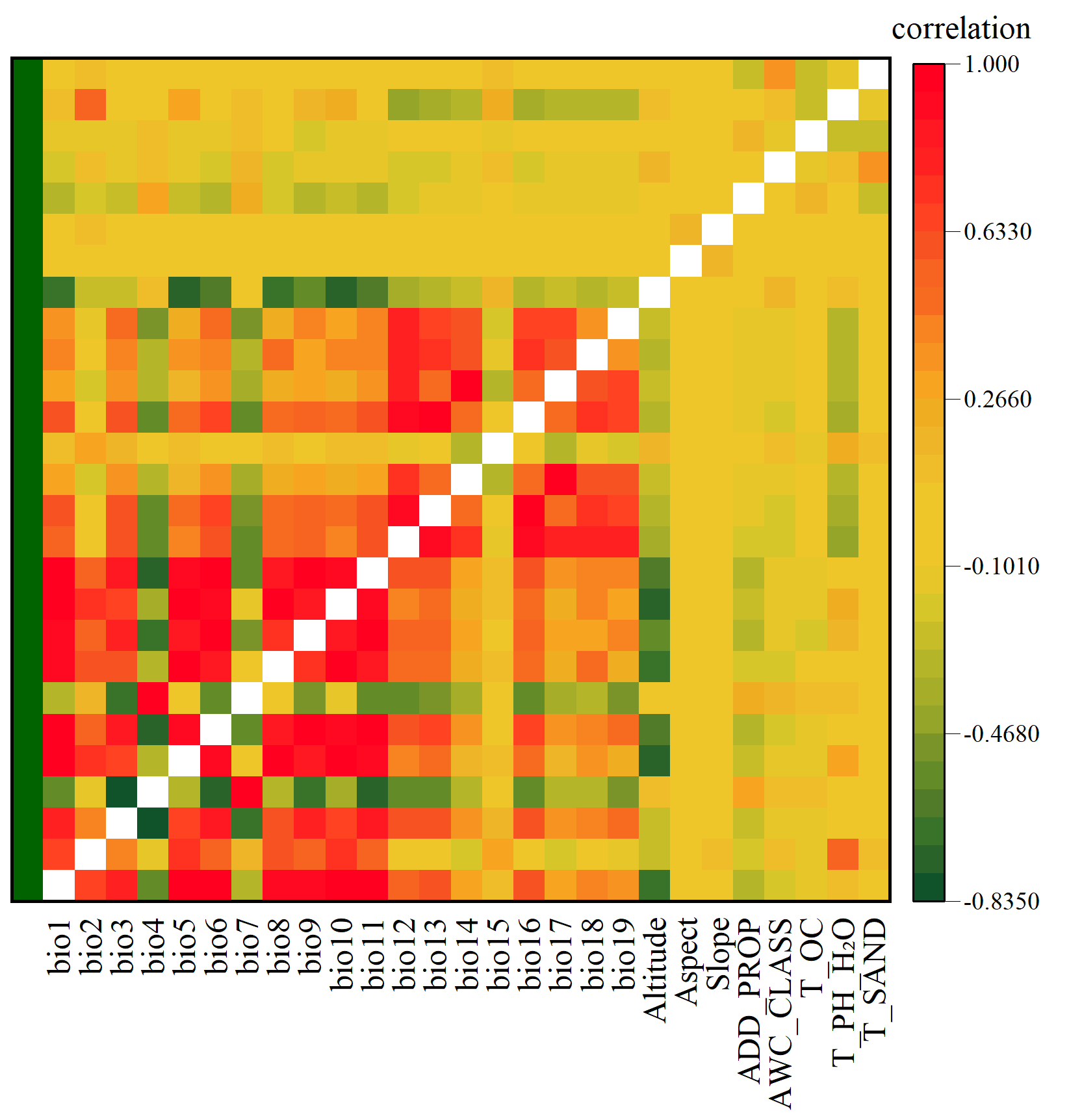


**Supplementary Figure 1.** The correlation among all used environmental variables.


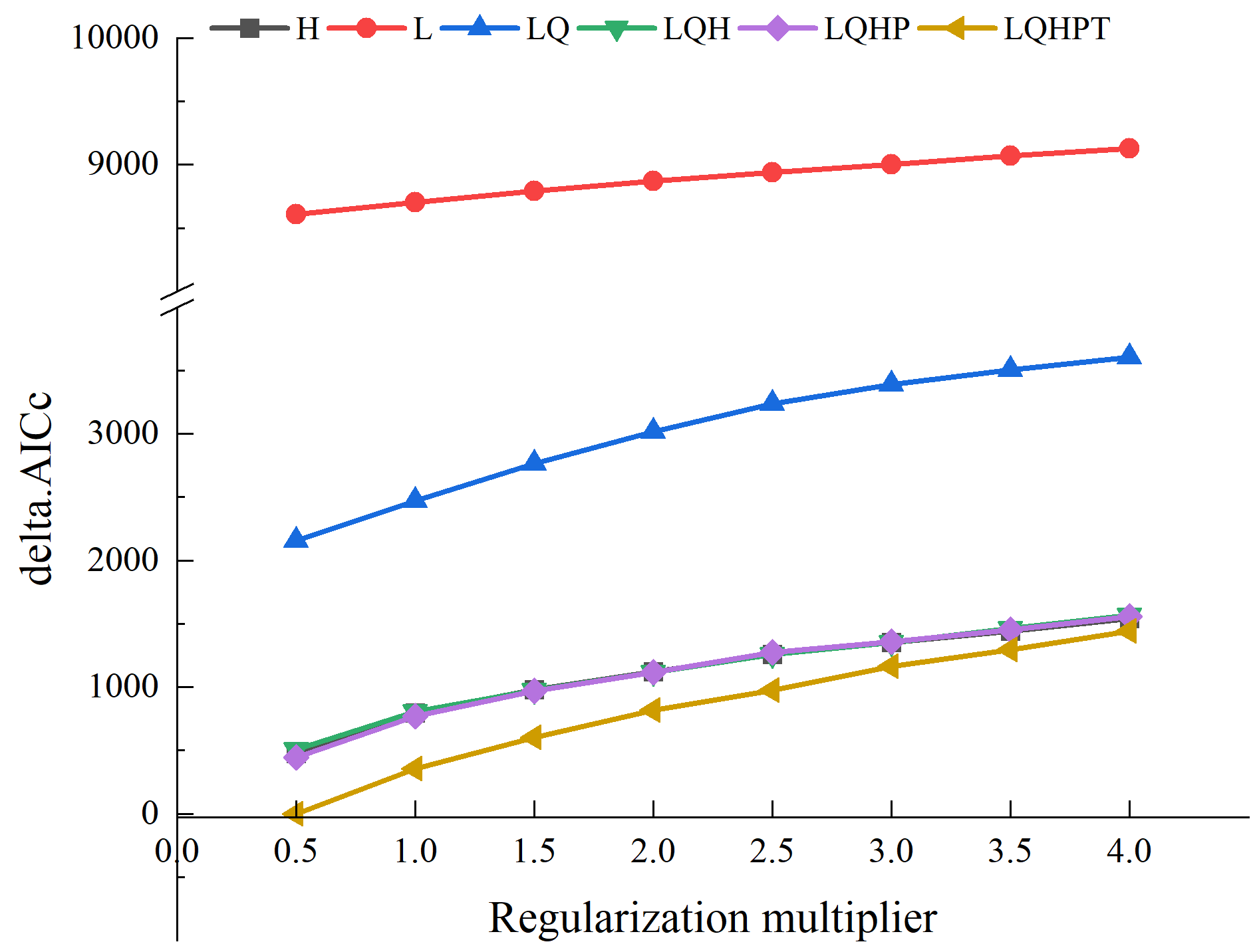


**Supplementary Figure 2.** Linear correlation of RM and ∆AICc. H: hinge , L: linear, Q: quadratic ,P: product, T: threshold. RM was set from 0.5 to 4. There are six FC. RM increased at an interval of 0.5. As shown above, ∆AICc was 0, indicating that LQHPT was the optimal FC.


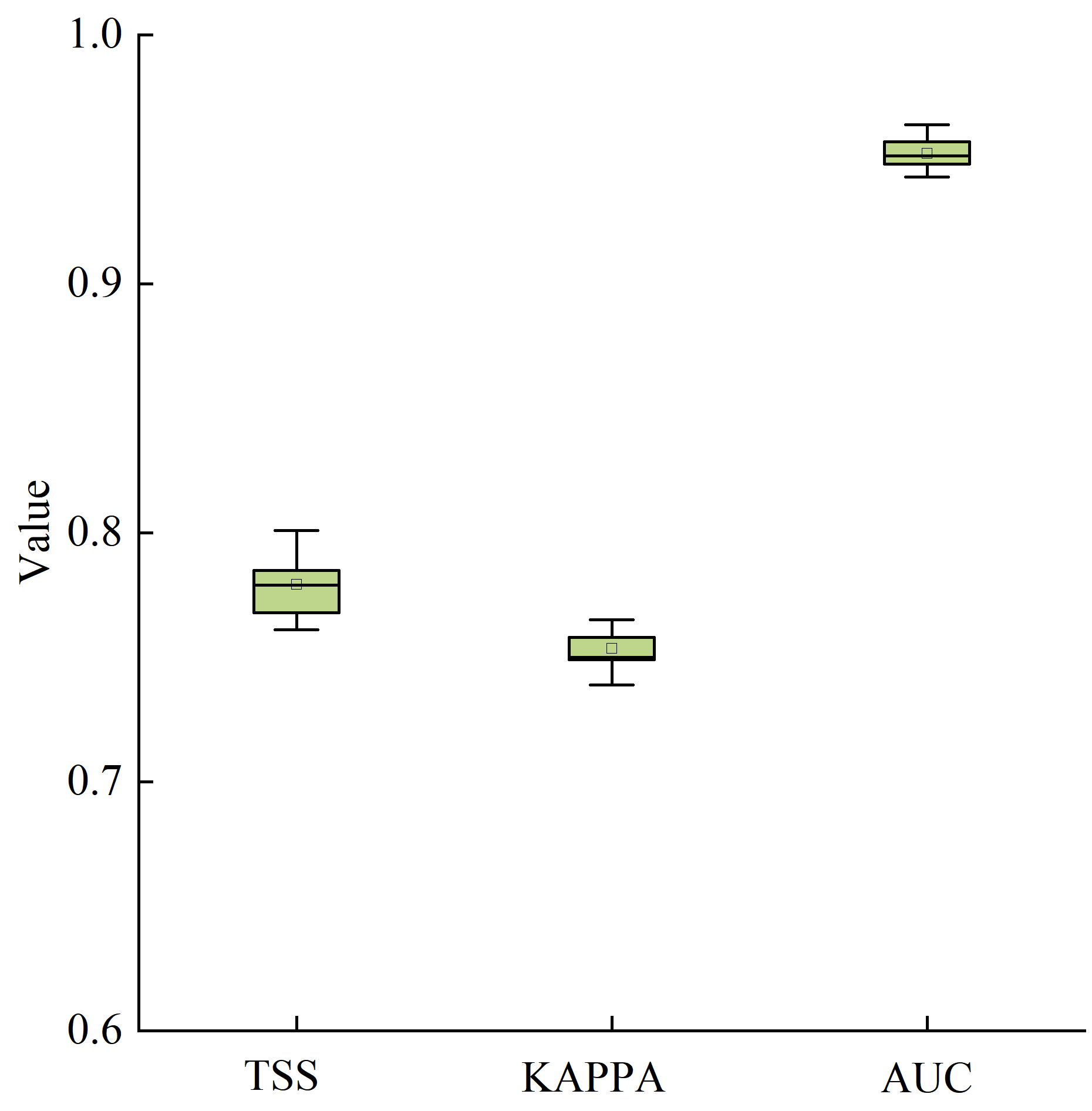


**Supplementary Figure 3.** TSS, KAAPA, and AUC value.


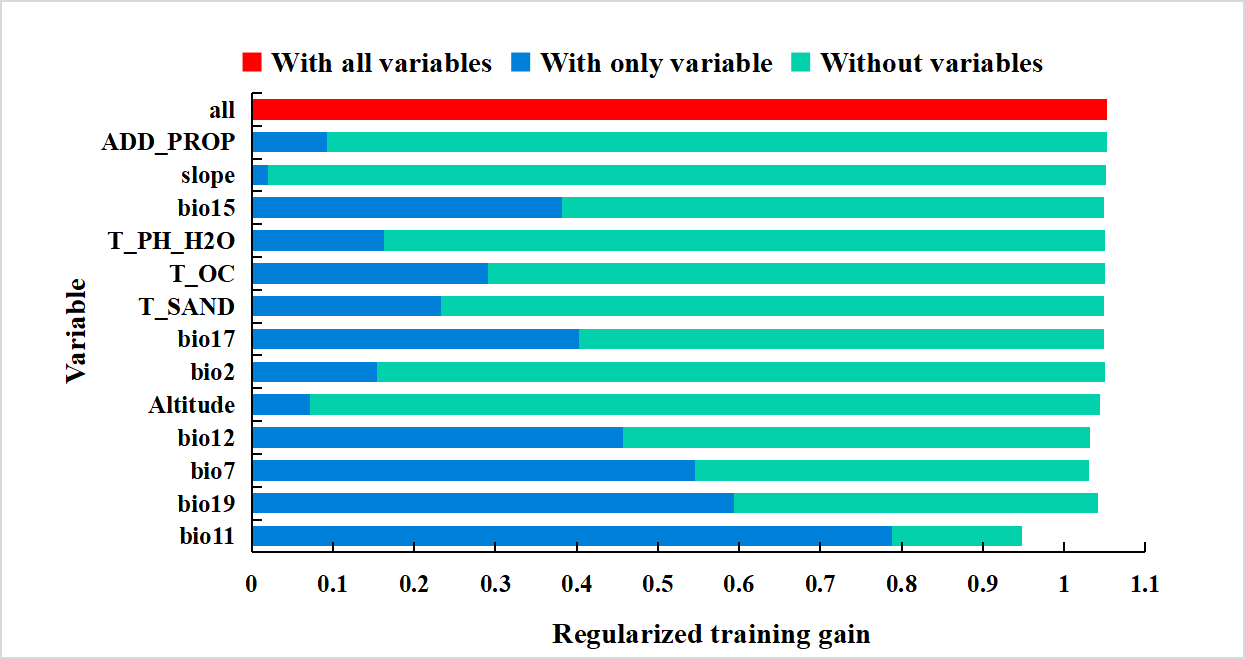


**Supplementary Figure 4.** Importance of environmental variables by “Jackknife method”.


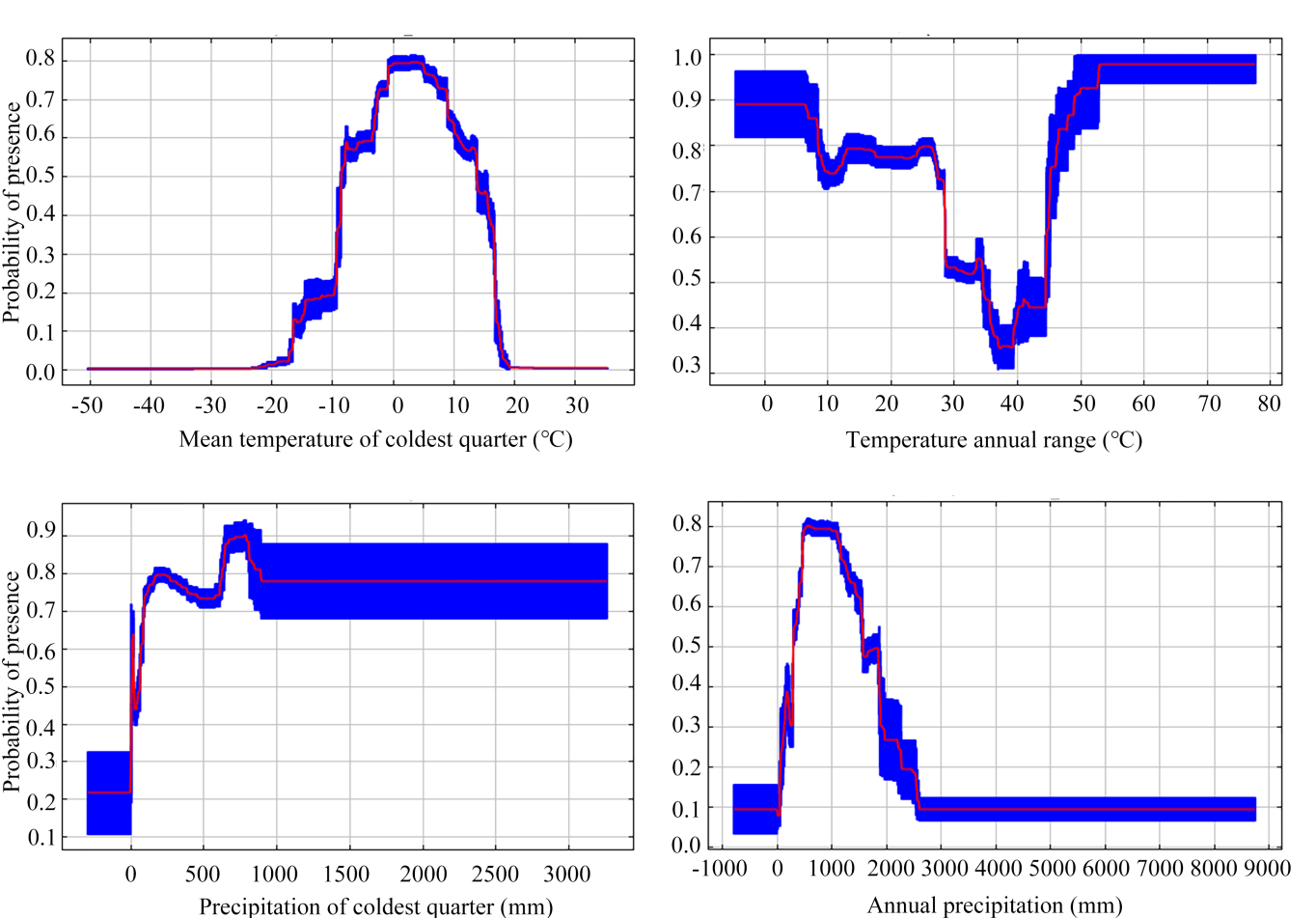


**Supplementary Figure 5.** Response curve of the most key environmental variables.


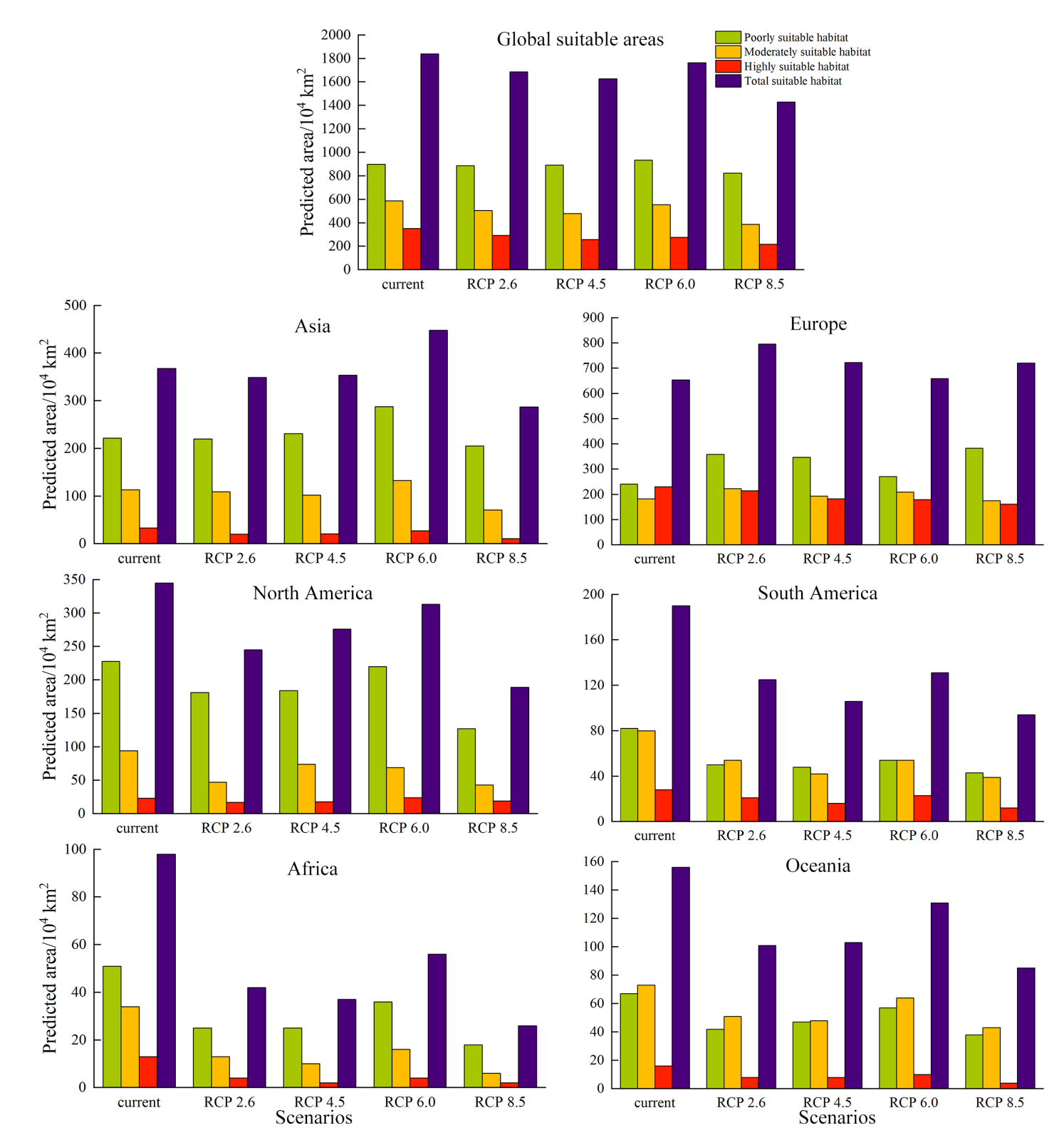


**Supplementary Figure 6.** Suitable areas of *Lolium temulentum* under the current and four future climate scenarios, across the globe and six continents (Asia, Europe, North America, South America, Africa, and Oceania)

## Supplementary Tables

**Supplementary Tables 1.** Environmental variables for the potential geographical distribution of *Lolium temulentum.*

| Variable | Description | Unit |
| --- | --- | --- |
| bio1 | Annual mean temperature | ℃ |
| bio2 | Mean diurnal temperature range | ℃ |
| bio3 | Isothermality (Bio2/Bio7)×100 | - |
| bio4 | Temperature seasonality (standard deviation×100) | ℃ |
| bio5 | Max temperature of warmest month | ℃ |
| bio6 | Min temperature of coldest month | ℃ |
| bio7 | Temperature annual range | ℃ |
| bio8 | Mean temperature of wettest quarter | ℃ |
| bio9 | Mean temperature of driest quarter | ℃ |
| bio10 | Mean temperature of warmest quarter | ℃ |
| bio11 | Mean temperature of coldest quarter | ℃ |
| bio12 | Annual precipitation | mm |
| bio13 | Precipitation of wettest month | mm |
| bio14 | precipitation of driest month | mm |
| bio15 | Precipitation seasonality (coeffcient of variation×1 | - |
| bio16 | Precipitation of wettest quarter | mm |
| bio17 | Precipitation of driest quarter | mm |
| bio18 | Precipitation of warmest quarter | mm |
| bio19 | Precipitation of coldest quarter | mm |
| Altitude | Altitude | m |
| Slope | Slope |  |
| Aspect | Aspect | % |
| ADD_PROP | Other properties (gelic, vertic, petric) |  |
| AWC_CLASS | Soil Available Water Content Range |  |
| T_OC | Topsoil Organic Carbon | % weight |
| T_PH_H_2_O | Topsoil pH (H_2_O) | -log(H^+^ ) |
| T_SAND | Topsoil Sand Fraction | % wt |

**Supplementary Tables 2.** Suitable areas of *Lolium temulentum* in each continent under the current and the RCP 2.6, RCP 4.5, RCP 6.0, and RCP 8.5 in the 2050s (× 10^4^ km^2^)*.*

|  | Poorly  suitable habitat | Moderately suitable habitat | Highly  suitable habitat | Total  suitable habitat |
| --- | --- | --- | --- | --- |
| Asia |  |  |  |  |
| Current | 222 | 113 | 33 | 368 |
| RCP 2.6 | 220 | 109 | 20 | 349 |
| RCP 4.5 | 231 | 102 | 21 | 354 |
| RCP 6.0 | 288 | 133 | 27 | 448 |
| RCP 8.5 | 205 | 71 | 11 | 287 |
| Europe |  |  |  |  |
| Current | 241 | 183 | 230 | 654 |
| RCP 2.6 | 359 | 223 | 214 | 796 |
| RCP 4.5 | 347 | 193 | 183 | 723 |
| RCP 6.0 | 271 | 209 | 179 | 659 |
| RCP 8.5 | 383 | 175 | 162 | 720 |
| North America |  |  |  |  |
| Current | 228 | 94 | 23 | 345 |
| RCP 2.6 | 181 | 47 | 17 | 245 |
| RCP 4.5 | 184 | 74 | 18 | 276 |
| RCP 6.0 | 220 | 69 | 24 | 313 |
| RCP 8.5 | 127 | 43 | 19 | 189 |
| South America |  |  |  |  |
| Current | 82 | 80 | 28 | 190 |
| RCP 2.6 | 50 | 54 | 21 | 125 |
| RCP 4.5 | 48 | 42 | 16 | 106 |
| RCP 6.0 | 54 | 54 | 23 | 131 |
| RCP 8.5 | 43 | 39 | 12 | 94 |
| Africa |  |  |  |  |
| Current | 51 | 34 | 13 | 98 |
| RCP 2.6 | 25 | 13 | 4 | 42 |
| RCP 4.5 | 25 | 10 | 2 | 37 |
| RCP 6.0 | 36 | 16 | 4 | 56 |
| RCP 8.5 | 18 | 6 | 2 | 26 |
| Oceania |  |  |  |  |
| Current | 67 | 73 | 16 | 156 |
| RCP 2.6 | 42 | 51 | 8 | 101 |
| RCP 4.5 | 47 | 48 | 8 | 103 |
| RCP 6.0 | 57 | 64 | 10 | 131 |
| RCP 8.5 | 38 | 43 | 4 | 85 |
